# Difference between Leisure and Work Contexts: The Roles of Perceived Enjoyment and Perceived Usefulness in Predicting Mobile Video Calling Use Acceptance

**DOI:** 10.3389/fpsyg.2017.00350

**Published:** 2017-03-08

**Authors:** Ronggang Zhou, Caihong Feng

**Affiliations:** School of Economics and Management, Beihang UniversityBeijing, China

**Keywords:** mobile video calling, technology acceptance model, perceived enjoyment, perceived usefulness, work context, leisure context

## Abstract

There is a rapidly growing body of literature on mobile video calling, which is a promising communication technology; however, little research has focused on user acceptance of mobile video calling, especially in different use contexts. This study explored factors (especially perceived enjoyment) influencing the intention of users to employ video calling in different contexts (a work and a leisure context) by applying the technology acceptance model (TAM) combined with the theory of planned behavior. The revised research model differentiated external factors (subjective norms and personal innovativeness) from internal factors (perceived usefulness, perceived ease of use (PEU), perceived enjoyment, and intention to use mobile video calling). In addition, the current study investigated predictors of perceived enjoyment across these two contexts. With the use of a structured questionnaire, participants were divided in two groups and completed self-report measures related to one context; a total of 386 student respondents’ responses were analyzed. The results indicated that users’ intentions were directly predicted by their perceived enjoyment of video calling (β ≥ 0.35) and the call’s perceived usefulness (β ≥ 0.27) and PEU (β = 0.13, only for the leisure context), which jointly explained at least 55.6% of the variance in use intention. In addition to the effects of these predictors on mobile video calling use acceptance, an assessment of the moderating effects of different contexts indicated that perceived enjoyment played a more important role in influencing intention for the leisure context, while perceived usefulness appeared to be more important for the work context. This study’s findings are important in that they provide strong support for the necessity of distinguishing among different types of contexts when predicting users’ intentions to use video calling. Furthermore, the results showed that perceived enjoyment was most significantly influenced by perceived usefulness (β ≥ 0.61), followed by PEU (β ≥ 0.13). In summary, the roles of core TAM variables (especially perceived enjoyment and perceived usefulness) and of external factors (subjective norms and personal innovativeness) differed between the leisure and work contexts. The implications of these findings are discussed.

## Introduction

Mobile value-added services (e.g., web games, chat rooms, e-mail, and online payments) have become an important resource that creates revenue for business providers. One of these companies’ aims is to attract new subscribers and retain old ones. With the development of telecommunication technologies (such as 4G and LTE) and smart phones, certain new and important mobile value-added services continue to attract people who use cell phones to communicate via video calling. For example, Juniper Research forecasted that video calling (i.e., Skype) users alone are expected to increase by over 130 million by 2018. This figure does not include messaging, push-to-talk or audio calling options (Nay, 2014). Recently, the Facebook Messenger app became a competitor in the video calling market. This app can be used for video calls, even those between an iOS user and an Android user. Facebook indicated that it had 600 million monthly Messenger users (Alan, 2015). In China, providers have made many attempts to encourage the use of mobile video calling, but the effect has been minor (Yang, 2014). In 2013, the Internet Data Center (IDC) predicted that the proportion of users who use video calling will not see rapid growth in China; an increase from 3.1% in 2013 to 4.9% in 2018 is expected. Video calling is not a common occurrence in the daily lives of most users; however, overall, Chinese Internet users are familiar with video communication via instant messaging services, such as QQ (Internet Data Center, 2013).

Although telecommunications firms in China have been committed to solving technical issues to offer better technical support for the use of video calling, why has this service not been more widely accepted? With the growing popularity of smart phones and the development of wireless network technology in mobile phones, those support technologies can be realized. Under these circumstances, video calling has naturally become a new opportunity for telecom service providers to generate revenue. Information and communication technology (ICT) firms are trying to capture a competitive edge and greater market share in video calling and related markets. However, it is unclear how they can attract customers. Consumer acceptance is probably the main factor driving the diffusion of video calling. Video calling is an attractive technology, as users can make video calls with friends and family during leisure time and use it for video conferencing during work hours. Therefore, reliably identifying and understanding factors affecting consumers’ behavioral intention to use this service in different contexts has become a key issue.

From the perspective of consumer demand, telecommunications providers have a vested interest in understanding the behavior of video call users. A significant body of theoretical research has focused on media, technology, and service acceptance behavior by applying the theory of reasoned action (TRA) (Ajzen and Fishbein, 1980), the theory of planned behavior (TPB) (Ajzen, 1991), and the technology acceptance model (TAM) (Davis, 1989). Although there is also a rich and rapidly growing body of literature on video calling, surprisingly little research has been conducted based on the TAM. This model can be used to investigate how life contexts (such as work or leisure contexts) affect users’ acceptance of new technologies, such as video calling services. Empirical studies are needed to explore the predictive effects of factors that are relevant to video calling acceptance behavior while considering different contexts. This study proposes a research model that integrates components of the TPB with the revised TAM to examine the effects of factors influencing acceptance behavior outcomes for video calling. Furthermore, this work compares the predictive effects of perceived usefulness and perceived enjoyment on the intention to use video calling services, in particular, including work contexts (i.e., work-related purposes) and leisure contexts (i.e., chat-related purposes). The outcomes should also benefit practitioners in the telecom sector by helping them to identify appropriate marketing strategies to increase the acceptance of video calling in the future.

## Literature Review and Research Hypotheses

In the current study, we used the TAM as a theoretical framework, adding belief elements from the TPB to examine users’ acceptance of video calling technology in leisure and work contexts. In the following sections, we review empirical studies focusing on user acceptance and then identify research hypotheses.

### Theoretical Models to Understanding Users’ Decision to Use Technology

#### Theories of Reasoned Action and Planned Behavior (TRA and TPB)

In terms of understanding users’ decision to use technology, several theoretical models have been widely applied in previous studies. The earlier related model of the TRA assumes that beliefs affect behavior; it is a well-known model for predicting the intention to perform a behavior based on an individual’s attitudinal and normative beliefs (Fishbein and Ajzen, 1975; Ajzen and Fishbein, 1980; Ajzen, 1985; Jin, 2014). Then, the TPB, an extension of the TRA, was proposed to accommodate new ways of identifying variables (Schifter and Ajzen, 1985; Ajzen, 1991). Both the TRA and the TPB have been used in various topics to investigate the influence of personal variables (e.g., subjective norms, individual traits, and perceived behavioral control) on behavioral intentions or decision behaviors, such as the acceptance of the World Wide Web (Klobas and Clyde, 2000) and the acceptance of mobile technology (Luarn and Lin, 2005). The two models, especially the TPB, have been employed in a wide range of behavioral disciplines and proven to be a successful model for understanding behavior in a variety of situations (Kang et al., 2006). Additionally, the TRA and TPB provided a theoretical foundation for the development of the TAM for investigating users’ decision to use technology or technology-related products.

#### Technology Acceptance Model (TAM)

Based on the TRA, Davis et al. (1989) developed the TAM to predict information technology acceptance and usage behavior. A variety of studies have confirmed that the TAM is applicable for example, the use of various technologies applications (Agarwal, 2000), such as the World Wide Web (Horst et al., 2007), electronic commerce (Chung et al., 2010), mobile devices (Lin and Liu, 2009), and technological implants (Pelegrín-Borondo et al., 2016). Among several applicable theoretical frameworks, the TAM may be the most suitable theory for studying users’ acceptance of new technology in the following aspects. (1) Its reliability has been demonstrated in various tests (Venkatesh and Davis, 2000; Jin, 2014). (2) Compared with other theoretical models used to explain consumer behavior and information technology adoption, the high generalizability and interactivity of the TAM facilitate proposing more valuable practical applications (Davis et al., 1989; Venkatesh and Davis, 2000; Juaneda-Ayensa et al., 2016). (3) In accordance with specific research aims, the TAM allows the inclusion of core variables (perceived usefulness, perceived ease of use (PEU), attitudes and intention to behave in a certain way, which is the major determinant of actual usage behavior) (Davis, 1989; Davis et al., 1989; Yi et al., 2006) and numerous extended measures (e.g., playfulness, subjective norms, innovativeness, and self-efficacy) for explaining ICT use (Igbaria et al., 1995; Karahanna et al., 1999; Agarwal and Karahanna, 2000; Venkatesh and Davis, 2000).

Having comprehensively considered the various theories, this study generally used the TAM as its basic theoretical foundation while including the beliefs of the revised TPB as external variables. Previous research has successfully integrated the TPB into the TAM to investigate technology acceptance behavior (Akman and Mishra, 2015). The current study aimed to investigate how perceived enjoyment and the two original TAM variables (i.e., perceived usefulness and PEU) influence users’ engagement in video calling in different contexts. Several studies have explored whether the belief-based variables of the TAM are mediators of its external variables (Venkatesh and Brown, 2001; Porter and Donthu, 2006); thus, this study also extended the above core TAM variable by adding external variables from the TPB, including subjective norms and personal innovativeness. The follow sections review these variables and issues.

### Effects of Components of TAM on Users’ Decision and Proposed Research Model

According to the TAM and the TPB, several important internal and external factors affect consumers’ intention to adopt video calling services: perceived usefulness, PEU, perceived enjoyment, subjective norms, and personal innovativeness. Furthermore, different contexts of use should be considered a crucial factor to be investigated.

#### Standard Variables: Perceived Usefulness, Perceived Ease of Use and Perceived Enjoyment

Perceived usefulness and PEU, as the TAM’s primary factors, determine users’ acceptance or rejection of ICT (Davis, 1989). Perceived usefulness is defined as “the degree to which a person believes that using the particular technology would enhance his/her job performance.” PEU is defined as “the extent to which a person believes that using a technology is free of effort.” Perceived usefulness and PEU jointly determine attitudes toward usage behavior or directly predict behavioral intention (Davis, 1989). Perceived usefulness also mediates the effect of PEU on behavioral intention. Perceived usefulness can predict the behavioral intention to use, which directly affects actual usage behavior. These two attitudinal factors (perceived usefulness and PEU) are always considered as extrinsic motivation for usage intention (Davis and Wiedenbeck, 2001). Since the original model of TAM was proposed, the effects of perceived usefulness and PEU on users’ acceptance of technology have been supported by a large body of research. From an instrumental perspective, performing a behavior is considered a means to achieve other goals or to gain other valued outcomes (Venkatesh, 2000). In this respect, the decision to use mobile video calling can be predicted by perceived usefulness.

However, previous studies have indicated that perceived usefulness and PEU cannot fully explain consumers’ behavioral intentions to use a new technology (Venkatesh and Davis, 2000; Chen et al., 2002; Bruner and Kumar, 2005; Liao and Tsou, 2009; Jin, 2014). Researchers have found that technology usage is affected by both extrinsic motivation (i.e., usefulness) and intrinsic motivation (i.e., enjoyment). Perceived enjoyment has been defined as “the extent to which the activity of using the computer is perceived to be enjoyable in its own right, apart from any performance consequences that may be anticipated” (Davis and Wiedenbeck, 2001). With more “hedonic” service or technology use acceptance addressed by TAM (e.g., Van der Heijden, 2004), perceived enjoyment has been confirmed to have a significant influence on users’ intention to use technology. A number of studies have indicated that enjoyment is a particularly powerful predictor of use decision for technologies such as Facebook (Quan-Haase and Young, 2010), Sina Weibo (Wang et al., 2016), mass media (Nabi and Krcmar, 2004; Ledbetter et al., 2016), the telephone (O’Keefe and Sulanowski, 1995), websites (Van der Heijden, 2003), online shopping (Childers et al., 2001), and social networking sites (Chuang et al., 2017). Although we found no research on mobile video calling use acceptance in terms of perceived enjoyment, some previous studies have addressed the issue for other interpersonal communication media or technologies, such as instant messaging (Li et al., 2005; Lu and Xu, 2006). Several theoretical perspectives assert that enjoyment “might be the most basic motivation to consume any communication media” (Sherry, 2004; Griffin et al., 2015). Lu and Xu (2006) proposed three additional variables based on the TAM, including an “immersion” experience, perceived enjoyment and privacy. Then, they conducted a study on instant communication usage behavior, which examined whether behavioral intention was a function of perceived usefulness, perceived enjoyment, “immersion” experience, privacy or attitude. They concluded that perceived enjoyment has a direct effect on behavioral intention. All these findings imply that the intrinsic motivational factor, enjoyment, may also play a significant role in acceptance of mobile value-added services, such as video calling.

Another important issue is related to the relationship among perceived enjoyment, perceived usefulness, and PEU. In the original version of the TAM, generally perceived usefulness has a more powerful effect than PEU in predicting behavioral use intention, and perceived usefulness also mediates the effect of PEU on user acceptance (Davis, 1989). Some previous studies intended to focus on the association among perceived enjoyment, perceived usefulness, and PEU (e.g., Davis et al., 1992; Van der Heijden, 2004; Sun and Zhang, 2006). However, no consistent results can be concluded based on previous studies. On the one hand, perceived enjoyment has been shown to have a significant influence on predicting the related constructs of perceived usefulness and PEU (e.g., Venkatesh, 2000; Teo and Noyes, 2011). On the other hand, some studies have indicated that perceived usefulness and PEU had a more significant effect on perceived enjoyment (e.g., Van der Heijden, 2004; Liao et al., 2008). Sun and Zhang (2006) reviewed the relevant literature and investigated the causal relationship between perceived enjoyment and PEU. Their findings indicated that the direction of perceived enjoyment influenced the PEU, which outweighed the opposing direction for utilitarian systems. However, when perceived enjoyment was considered to explain users’ technology use decisions, some researchers removed the construct of PEU, and their findings tended to support that the influence of perceived enjoyment on behavioral intention outweighs the predictive effect of perceived usefulness on behavioral intention (e.g., Verkasalo et al., 2010; Lin and Lu, 2011). Although mobile video calling can be used for both work and leisure goals, we tend to consider it as an individual hedonic service. Thus, the influences of perceived usefulness and PEU on perceived enjoyment were examined.

Collectively, regarding mobile video calling use acceptance, the above concerns led to the following hypotheses:

H1. Perceived usefulness would have a positive influence on the perceived enjoyment of video calling usage in addition to having a direct impact on users’ behavioral intention to use mobile video calling.
H2. Perceived ease of use would have a positive influence on the perceived enjoyment of video calling usage.
H3. Perceived enjoyment would have a positive influence on users’ behavioral intention to adopt mobile video calling.
H4. Perceived usefulness and perceived enjoyment would jointly determine the behavioral intention to use mobile video calling.

#### Extended Variables: Subjective Norms and Perceived Innovativeness

According to the TRA and TPB, subjective norms reflect how consumers are affected by their perception that those who are important to them think that they should (or should not) perform a given behavior (Warner and DeFleur, 1969; Fishbein and Ajzen, 1973; Schofield, 1975; Hasan et al., 2016; Wei et al., 2016). As the common component of the TRA and the TPB, the positive effect of subjective norms on users’ behavioral intentions has been validated by numerous studies. For example, Peace et al. (2003) studied software piracy technology intentions using a model based on the TRA, the TPB, expected utility theory and deterrence theory; they found that subjective norms were a significant predictor of the intention to illegally copy software technology. Venkatesh et al. (2003) used a unified view to consider user acceptance of information technology, and their research suggested that subjective norms significantly influenced behavioral intentions in mandatory environments (such as work contexts). Hsu and Lu’s (2004) research also indicated that subjective norms and attitudes together explained 80% of variance in network game technology users’ behavioral intentions. These cumulative results suggested that users perceiving much greater approval and support from the people around them can increase user acceptance decisions.

Another important individual variable is personal innovativeness, which is defined as an individual’s willingness to try out any new technology (Agarwal and Prasad, 1998). Personal innovativeness was found to also positively affect individuals’ perceptions of new information technologies (Lewis et al., 2003; López-Nicolás et al., 2008). Some previous studies indicated that personal innovativeness has an influence on users’ attitudes toward accepting new technologies. Rogers (1971) believed that personal innovativeness could predict users’ attitudes toward acceptance of new technology; users with a high level of personal innovativeness were usually earlier to accept new technology. Herrero Crespo and Rodríguez del Bosque (2008) identified effects of personal innovativeness on the acceptance of e-commerce. Their results indicated that electronic commerce acceptance is determined by attitudes (or emotional evaluations) toward the system, subjective norms and personal innovativeness in the domain of information technology. Yang et al. (2012) argued that the ability of personal innovativeness to predict users’ acceptance of new technology is influenced by the environment. Regarding the association between perceived enjoyment and personal innovativeness, some people may have doubts and resistance to using new technology, while others can perceive enjoyment from new technology. Those with a high level of personal innovativeness may tend to more easily derive enjoyment and satisfaction from using new technology. However, the relationship between personal innovativeness and perceived enjoyment needs more understanding. Therefore, in this study, we considered the effects of personal innovativeness on the acceptance of video calling technology.

Since the role of perceived enjoyment in the acceptance of video calling was investigated in this study, the predictive effects of subjective norms and personal innovativeness on using this mobile service may also be moderated through perceived enjoyment. Thus, when considered as a whole, TAM-based studies indicate that the above-mentioned variables from the TAM and the TPB should significantly affect consumer acceptance and usage of video calling. That is, the external variables are strongly interrelated according to the TAM. The corresponding hypotheses regarding subjective norms and personal innovativeness that were tested in this study are as follows:

H5. Subjective norms positively would affect users’ perceived enjoyment and use decisions regarding mobile video calling.
H6. Personal innovativeness positively would affect users’ perceived enjoyment and use decisions regarding mobile video calling.

### Influence of Use Context on Video Calling Use Decisions

Most previous empirical studies based on the TAM were based on voluntary contexts, and all the participants using the new technology were voluntarily participating in leisure contexts (Davis et al., 1989). However, whether their conclusions were applicable to the condition of forced work remained to be explored.

The core variables (e.g., perceived usefulness, PEU, and perceived enjoyment) in the TAM are consistent with basic measures of user experience. For example, to consolidate the definitions in usability community, the International Organization for Standardization (ISO) defined usability as “the extent to which a product can be used by specified users to achieve specified goals with effectiveness, efficiency and satisfaction in a specified context of use.” That is, user perception and acceptance of technology can mean different things to different people in different specific use contexts. In an earlier study, Davis et al. (1989) also indicated the importance of use contexts with the use of the TAM framework. They explored users’ acceptance of WriteOne, which is a type of human-computer interaction technology, and noted a limitation of their study, i.e., all users were in a leisure context in which there was no compulsion to use the technology. They admitted that they ignored the context variable and emphasized the necessity of discussing the influence of different contexts when exploring users’ acceptance of new technologies. After that, many extended TAM researchers investigating users’ acceptance of new technologies began to take usage contexts into consideration (Moore and Benbasat, 1991; Hartwick and Barki, 1994; Van der Heijden, 2004).

As a motivational variable, users’ enjoyment perception tends to be more dependent on use context. Some previous studies considered perceived enjoyment to be a focal aspect of entertainment media usage because individuals consume these media primarily to seek fun or pleasure (Vorderer et al., 2004; Pe-Than et al., 2014). However, beyond the entertainment context, perceived enjoyment was also found to have a substantial impact on users’ behavioral intentions regarding task-oriented applications, such as online information sharing (Li et al., 2005; Kim et al., 2009; Pe-Than et al., 2014). Bruner and Kumar (2005) focused on Palm Internet equipment in an empirical study employing the revised TAM model in the consumer context. The influencing factors were divided into two types: practical factors and interest factors. The results indicated that in leisure contexts (differing from the results for work-related contexts), the effect of perceived enjoyment on behavioral intention was more significant than that of perceived usefulness, which had no significance. In fact, in their research on acceptance of the World Wide Web, Moon and Kim (2001) assert that attitudes can be influenced by situational factors and the interaction between an individual and a given situation.

Clearly, the motivations and feelings of users who use these technologies in different contexts differ. Correspondingly, the degree to which different factors influence customers’ acceptance of these technologies will also vary. As a new type of information communication technology, video calling has characteristics oriented toward both entertainment and work. Additionally, potential users can either use cell phones to make video calls with friends and family during their leisure time or for video conferencing during work time, implying that the degree to which the varying factors affect users’ behavioral intentions may differ in different contexts. However, few previous studies related to video calling have focused on investigating how different situations affect users’ video calling acceptance based on the TAM. Therefore, this study proposed considering the different contexts (work contexts and leisure contexts) to investigate the ways in which different contexts influence users’ acceptance of video calling technology. The corresponding hypothesis was proposed as follows:

H7. The degree to which perceived enjoyment affects behavioral intentions to use mobile video calling would vary in different user contexts, i.e., work contexts and leisure contexts.

**FIGURE 1:**
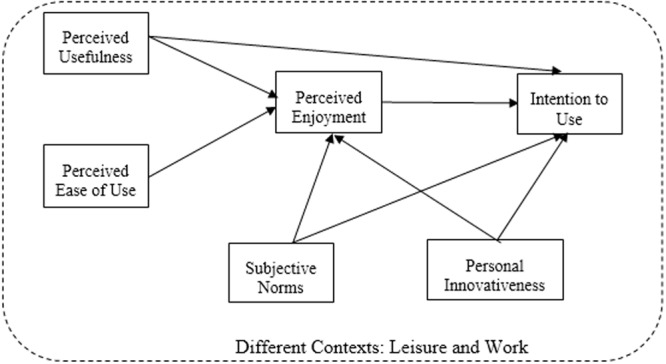
**Proposed research model**.

Thus, these proposed hypotheses generated the research model described in **Figure 1**.

## Materials and Methods

### Materials

#### Video Calling Use Context

Following a method used in previous studies adopting the TPB that encourages respondents to answer questions in a relatively natural way, two potential use contexts were generated, both including the same written introduction regarding video calling services. One context depicted a situation in which the respondents use video calling to contact a colleague for work purposes. The second context depicted a situation in which the respondents used video calling to contact a friend for leisure purposes.

The written introduction read as follows:

Since the issuing of 4G licenses and the completion of 4G networks, 4G mobile value-added services have become increasingly popular, including “video calling,” which is one of the typical services offered by telecommunications businesses. As the name suggests, cell phone video calling services enable both sides of the conversation to see each other smoothly and clearly in real time. Currently, when using a mobile phone to make video calls, both sides need to place a cell phone in front of them so that the phone’s camera can relay images of both users.

The contexts were as follows:

• Work Context
Suppose you currently have a position in a company located in a city that has established a 4G network and that you are currently using a 4G mobile phone that is able to support video calls. It is a weekday morning, and you are working. You need to use the phone to call a colleague to address a work-related issue. Your colleague’s cell phone also supports video calls. When you call, you can choose conventional talk mode (i.e., non-video call) or video call mode.
• Leisure Context
Suppose you currently have a position in a company located in a city that has established a 4G network and that you are currently using a 4G mobile phone that is able to support video calls. It is a weekend morning, and you are looking over photos you have previously taken. At this point, you remember that it is time for you to contact a friend, and you are ready to use the cell phone to call him or her to say hello. Your friend’s cell phone also supports video calls. When you call, you can choose conventional talk mode (i.e., non-video call) or video call mode.

#### Questionnaire

A self-administered questionnaire was developed based on existing scales, using minor wording modifications to fit items into the two different contexts. Following each context, and in line with previous studies on mobile service use decisions, participants responded to a series of items constructed based on an extended TAM questionnaire (i.e., behavioral intention, perceived usefulness, perceived enjoyment, and PEU). All items designed for the above measures were presented to the respondents in random order. Following the context-specific questions, respondents responded to questions about subjective norms and personal innovativeness (again, items were presented in random order), and they completed several demographic measures (e.g., age and gender). Respondents rated each item measuring the standard and extended components of the TAM on 7-point unipolar scales ranging from 1 (a negatively scored statement) to 7 (a positively scored statement).

##### Behavioral intention

Behavioral intention was assessed by calculating the mean score of the following four items (Davis, 1989; Venkatesh and Davis, 2000; Chen and Chen, 2009): “In the future, the likelihood that I will use video calling for this situation is (very unlikely to very likely)” (BI1); “In this context, I would expect to use video calling” (very unlikely to very likely) (BI2); “In this context, the likelihood that I will use video calling is (very unlikely to very likely)” (BI3); and “In the future, I will use video calling for this situation” (very unlikely to very likely) (BI4).

##### Perceived usefulness

Following a method used in previous studies (Davis, 1989; Venkatesh and Davis, 2000; Chen and Chen, 2009), the mean score of the following three items was used to measure perceived usefulness: “In this context, using video calling would help me to communicate with others” (strongly disagree to strongly agree) (PU1); “In this context, using video calling would help me to improve the efficiency of mobile communication” (strongly disagree to strongly agree) (PU2); and “In this context, using video calling is useful for me” (strongly disagree to strongly agree) (PU3).

##### Perceived enjoyment

Previous studies have also examined the perceived enjoyment of the use of mobile-data services. In line with these studies (Ragheb and Beard, 1982; Van der Heijden, 2003, 2004), the mean score of the following three items was used as a measure of perceived enjoyment: “In this context, the use of video calling provides me with enjoyment” (very unlikely to very likely) (PE1); “In this context, the use of video calling would be a good experience for me” (strongly disagree to strongly agree) (PE2); and “In this context, using video calling is pleasurable” (strongly disagree to strongly agree) (PE3).

##### Perceived ease of use

In line with previous studies using the TAM framework (Davis, 1989; Venkatesh and Davis, 2000; Chen and Chen, 2009), a direct aggregate measure was used to assess PEU. The mean score of the following three items was used as a measure of PEU: “In this context, it would be easy for me to become skilled at using video calling” (strongly disagree to strongly agree) (PEU1); “In this context, learning to use video calling would be easy for me” (strongly disagree to strongly agree) (PEU2); and “In this context, the use of video calling is (very difficult to very easy) for me” (PEU3).

##### Subjective norms

Previous studies have also examined subjective norms. In line with these studies (Taylor and Todd, 1995; Agarwal and Prasad, 1998), the mean score of the following two items was used as a measure of subjective norms: “In this context, people who influence my behavior would think that I should use video calling” (very unlikely to very likely) (SN1) and “In this context, I am expected to use video calling by the other person on the phone” (very unlikely to very likely) (SN2).

##### Personal innovativeness

Following a method used in previous studies (Hurt et al., 1977; Agarwal and Prasad, 1998), the mean score of the following three items was used to measure personal innovativeness: “If I heard about a new information technology, I would look for ways to experiment with it” (very unlikely to very likely) (PI1); “Among my peers, I am usually the first to try out new information technologies” (very unlikely to very likely) (PI2); and “I like to experiment with new information technologies” (very unlikely to very likely) (PI3).

##### Demographic measures

Demographic information, including age, gender, city of residence, specialized subject of study, service provider, and expenditure on mobile services per month, was also collected.

### Participants and Data Collection

In terms of gender and city of residence, the respondents were approximately balanced. A total of 386 students who were potential users of video calling participated in the study; however, 18 (4.7%) were eliminated due to incomplete or non-sensical responses. The subjects of the survey were university students from Beijing and Beihai, China. The two context-specific questionnaires were randomly distributed to the participants. Nearly half of the respondents from each city were asked to complete the questionnaire about the work context, and the other half from each city were asked to complete the questionnaire about the leisure context. Among the 368 respondents who provided valid responses, 182 respondents answered the questions regarding the work context (98 and 84 students were sampled from Beijing and Beihai, respectively), and the remaining 186 respondents answered the questions regarding the leisure context (101 and 85 students were sampled from Beijing and Beihai, respectively). Thus, the responses to each context-specific questionnaire were nearly equal. For the 368 valid responses, the subjects were between the ages of 17 and 26 years, with an average age of 20.9 years (*SD* = 1.5); 188 respondents were male (51.1%), and 180 were female (48.9%). In terms of specialized subject of study, 19.6% of the respondents reported that they specialized in human and social sciences; those studying some type of art accounted for only 3.3%. Most of the respondents specialized in management (38.6%) or science and technology (37.8%). Regarding service providers, most subjects reported using China Mobile (90.5%), while China Unicom and China Telecom represented only 4.6 and 3.8% of reported providers, respectively. In terms of expenditure on mobile services per month, almost 12.8% of users spent less than 30 Yuan; 34.8% spent between 30 and 50 Yuan; 26.9% spent between 31 and 70 Yuan; and the remainder (25.5%) spent more than 70 Yuan.

Each respondent was approached randomly by a trained interviewer at a campus location, such as a library or classroom. The study protocol was approved by the Human Research Ethics Committee in School of Economics and Management at Beihang University. All the respondents were asked to read a written introduction to the research and the format of the questionnaire, and they then completed an informed consent form, followed by the questionnaires. The respondents were assured that their participation was voluntary and that their responses would be anonymous. For those who agreed to participate, the questionnaire took approximately 10 min to complete.

## Results and Analysis

### Measurement Model

To analyze the measurement validity, we used AMOS 21.0 to examine the core TAM components in the research model, i.e., a four-factor structure including behavioral intention, perceived enjoyment, perceived usefulness, and PEU. Following similar, previously conducted studies (e.g., Jung et al., 2009), seven common model-fit measures were used to estimate the measurement model’s fit. As shown in **Table 1**, all of the model-fit index values satisfied or exceeded their respective common acceptance levels. Therefore, it was concluded that the measurement model had a good fit with the collected data.

**Table 1:** Fit indices for the measurement model.

Fit indices	Recommended	Result
	value	
χ^2^/d. f. (chi-square/degrees of freedom)	<3	2.314
GFI (goodness of fit index)	>0.9	0.948
RMSEA (root mean square error of approximation)	<0.08	0.060
RMR (root mean square residual)	<0.08	0.052
NFI (normed fit index)	>0.9	0.950
NNFI (non-normed fit index)	>0.9	0.961
CFI (comparative fit index)	>0.9	0.971

In addition to the model fit, we tested the validity and reliability of the measurements by verifying the convergent and discriminant validity of the core scales. As shown in **Table 2**, according to Tabachnick and Fidell (2007), the questionnaire items had high levels of convergent validity because the item loadings ranged from 0.71 to 0.86 and were all higher than 0.55. The higher the loading value is, the more accurate the item is as a measurement of the construct. A loading value above 0.55 is acceptable for interpreting the construct (Comrey and Lee, 1992). In addition, the lowest average variance extracted (AVE) among all the components was 0.588, which exceeded the requirement of 0.50 and demonstrated that the data had good convergent validity.

**Table 2:** Standardized factor loadings, SMC and CR for the TAM core variable questionnaires.

Construct	Item	Item loading	SMC	CR	AVE
Behavioral intention	BI1	0.728	0.531	0.866	0.618
	BI2	0.860	0.740		
	BI3	0.807	0.651		
	BI4	0.743	0.552		
Perceived enjoyment	PE1	0.716	0.512	0.827	0.615
	PE2	0.810	0.656		
	PE3	0.822	0.676		
Perceived usefulness	PU1	0.730	0.532	0.810	0.588
	PU2	0.750	0.563		
	PU3	0.817	0.667		
Perceived ease of use	PEU1	0.710	0.504	0.824	0.611
	PEU2	0.847	0.717		
	PEU3	0.781	0.610		

According to Fornell and Larcker (1981), if the AVE value is above 0.50 and the composite reliability (CR) value is above 0.70, then the reliability is acceptable. **Table 2** shows that all the squared multiple correlations (SMCs) of the measured variables were higher than the criterion (0.50) and that the CR values for all the constructs were above the recommended level of 0.70. These results indicate that the scales had good reliability. Fornell and Larcker (1981) also suggested that discriminant validity can be verified when the square root of the AVE (the diagonal elements in **Table 3**) for each construct is higher than its correlations with the other constructs. **Table 3** shows that all of the square roots of the AVE for each construct were higher than the correlations, indicating that the discriminant validity was acceptable. Overall, the validity and reliability of the questionnaire were acceptable.

**Table 3:** Descriptive statistics and zero-order correlations between the study variables: work and leisure contexts.

Variable	1	2	3	4	5	6	Gender^a^	Mean	*SD*
Work context (*n* = 182)									
(1) Behavioral intention	(0.87)	0.68^∗∗∗^	0.70^∗∗∗^	0.34^∗∗∗^	0.28^∗∗∗^	0.23^∗∗^	0.01	4.71	1.06
(2) Perceived enjoyment		(0.81)	0.73^∗∗∗^	0.39^∗∗∗^	0.36^∗∗∗^	0.22^∗∗^	-0.04	4.74	1.02
(3) Perceived usefulness			(0.80)	0.33^∗∗∗^	0.28^∗∗∗^	0.19	-0.01	4.64	1.06
(4) Perceived ease of use				(0.77)	0.35^∗∗∗^	0.27^∗∗∗^	-0.19^∗∗^	4.98	0.85
(5) Subjective norms					(0.68)	0.22^∗∗^	0.02	4.99	0.95
(6) Personal innovativeness						(0.70)	-0.11	4.52	0.98
Leisure context (*n* = 186)									
(1) Behavioral intention	(0.86)	0.73^∗∗∗^	0.68^∗∗∗^	0.49^∗∗∗^	0.50^∗∗∗^	0.45^∗∗∗^	-0.09	4.94	1.02
(2) Perceived enjoyment		(0.83)	0.77^∗∗∗^	0.53^∗∗∗^	0.45^∗∗∗^	0.42^∗∗∗^	-0.11	5.03	0.97
(3) Perceived usefulness			(0.82)	0.45^∗∗∗^	0.38^∗∗∗^	0.38^∗∗∗^	-0.10	4.82	1.02
(4) Perceived ease of use				(0.85)	0.46^∗∗∗^	0.34^∗∗∗^	-0.10	5.21	0.97
(5) Subjective norms					(0.75)	0.28^∗∗∗^	-0.09	5.08	1.07
(6) Personal innovativeness						(0.77)	-0.11	4.34	1.07

*Internal consistency estimates (Cronbach’s alpha) are in parentheses on the diagonal. ^a^Gender (1 = male, 2 = female). ^∗^*p* < 0.05, ^∗∗^*p* < 0.01, ^∗∗∗^*p* < 0.001.*

### Descriptive Statistics and Analysis

The means and zero-order correlation coefficients for the various measures, organized by context type (work or leisure), are shown in **Table 3**. Regarding the different contexts considered overall, the means of the scales indicate that respondents had a slightly positive intention to use video calling (*M* = 4.83), perceived the behavior to be somewhat enjoyable (*M* = 4.89), perceived this behavior as being of moderate usefulness (*M* = 4.73), perceived that the service should be easy to use (*M* = 5.10), felt social approval from important others (*M* = 5.04), and reported a medium degree of personal innovativeness (*M* = 4.43). All of the research model variables were found to positively correlate with each other in both situations. Gender was not associated with most of study variables, with the exception of negatively correlating with PEU in the work context.

To validate the study’s hypotheses, one-way analyses of variance were used to test whether the intention to use, along with other TAM variables, differed between the contexts (work and leisure). Compared with the work context, respondents were more likely to make a video call [*F*(1,367) = 4.51, *p* < 0.05, η^2^ = 0.01], perceived that the technology should be more enjoyable [*F*(1,367) = 7.56, *p* < 0.05, η^2^ = 0.02] and perceived that video calling should be easier to use [*F*(1,367) = 5.69, *p* < 0.05, η^2^ = 0.02] in the leisure context. There were no significant differences in perceived usefulness [*F*(1,367) = 2.96, *p* > 0.05, η^2^ = 0.01], subjective norms [*F*(1,367) = 2.78, *p* > 0.05, η^2^ = 0.01], or personal innovativeness [*F*(1,367) = 0.71, *p* > 0.05, η^2^ = 0.002] between the work and leisure contexts.

### Predicting Users’ Intention to Use Video Calling

The results reflecting the relationship between the standard TAM components and behavioral intention (obtained by regressing the predictive components on intention to use mobile video calling) are displayed in **Table 4**. This table shows the results of a series of hierarchical multiple linear regression analyses that were used to assess, for each context, the contributions of standard and extended components of the TAM to the prediction of behavioral intention. Correlational analyses and analyses of variance indicated that gender was not a significant variable for behavioral intention; therefore, it was not included as a predictor. For each of the two contexts, the key predictors of respondents’ intention to use the service were identified by first regressing the original TAM components (perceived usefulness and PEU) on behavioral intention, and then perceived enjoyment was added to the regression model. In the last step, the extended TAM variables (subjective norms and personal innovativeness) were added to the three-step hierarchical regression analysis. In this way, it was possible to assess the predictive utility of each variable after controlling for the influence of other variables.

**Table 4:** Hierarchical regression analyses: predicting intention to use mobile video calling.

Step and predictor	Leisure context (*n* = 186)			Work context (*n* = 182)		
	Step 1 β	Step 2 β	Step 3 β	Step 1 β	Step 2 β	Step 3 β
(1) Perceived ease of use	0.23^∗∗∗^	0.13^∗^	0.06	0.12^∗^	0.06	0.04
Perceived usefulness	0.57^∗∗∗^	0.27^∗∗∗^	0.25^∗∗^	0.66^∗∗∗^	0.43^∗∗∗^	0.42^∗∗∗^
(2) Perceived enjoyment		0.45^∗∗∗^	0.38^∗∗∗^		0.35^∗∗∗^	0.34^∗∗∗^
(3) Subjective norms			0.18^∗∗^			0.01
Personal innovativeness			0.13^∗^			0.06
*R*^2^	0.502	0.577	0.615	0.501	0.556	0.560
Δ*R*^2^	0.502	0.075	0.038	0.501	0.055	0.004
*F*_change_	92.23^∗∗∗^	32.40^∗∗∗^	8.92^∗∗∗^	89.96^∗∗∗^	21.98^∗∗∗^	0.73
*Degree of change freedom*	(2,183)	(1,182)	(2,180)	(2,179)	(1,178)	(2,176)

*^∗^*p* < 0.05, ^∗∗^*p* < 0.01, ^∗∗∗^*p* < 0.001.*

Considering the leisure context, in step 1, the two original TAM predictors (i.e., perceived usefulness and PEU) were able to explain 50.20% of the variance in behavioral intentions [*F*(2,183) = 92.23, *p* < 0.001], with both original predictors having a significant influence on predicting the intention to use video calling. In step 3, perceived enjoyment, when added to the regression analysis, was able to explain an additional 7.5% of the variance, resulting in a significant increase to 57.7% [*F*_change_ (1,182) = 32.40, *p* < 0.001], and all three TAM variables emerged as significant predictors (especially perceived enjoyment and perceived usefulness). In step 3, the extended TAM variables were able to explain an additional 3.8% of the variance in using video calling [*F*_change_ (2,180) = 8.92, *p* < 0.001], with subjective norms and personal innovativeness having a moderately limited but statistically significant independent effect, along with perceived enjoyment and perceived usefulness. In this step, the influence of PEU on behavioral intention disappeared.

In the work context, in step 1, the original TAM variables were able to explain 50.1% of the variance in mobile video calling [*F*(2,179) = 89.96, *p* < 0.001], with both variables (especially perceived usefulness) emerging as significant predictors. When added to the regression analysis in step 2, perceived enjoyment resulted in a substantial increase to 55.6% in the variance in using mobile video calling [Δ*R*^2^ = 5.5%, *F*_change_ (1,178) = 21.98, *p* < 0.001], with perceived enjoyment and perceived usefulness emerging as very significant predictors. The effect of PEU weakened to the point of non-significance. In step 3, when the extended TAM variables were added to the regression equation, they were able to explain only an additional 0.40% of the variance [*F*(2,176) = 0.73, *p* > 0.05]. Subjective norms and personal innovativeness did not emerge as significant predictors along with PEU.

Overall, in the context of work, perceived usefulness was a strong determinant of intention to use video calling, and perceived enjoyment was a significant secondary determinant. By contrast, for potential leisure usage, perceived enjoyment was the strongest predictor and perceived usefulness become a secondary predictor. In addition, the context mediated the predictive power of PEU and the two extended variables (subjective norms and personal innovativeness), which tended to have a moderate or small independent effect on the intention to use video calling in the leisure situation only.

### Predicting Users’ Perceived Enjoyment of Video Calling

To address the effects of the two original TAM variables and the two extended TAM variables on perceived enjoyment and to test the corresponding hypothesis, a two-step hierarchical regression analysis was conducted to investigate the utility of the predictors, especially perceived usefulness and PEU, for perceived enjoyment. For each of the two contexts, perceived usefulness and PEU were entered in step 1, and the extended variables of subjective norms and personal innovativeness were added in step 2. By controlling for the influence of other variables, this approach allowed us to test the associations between perceived enjoyment and other study variables. The results are summarized in **Table 5**.

**Table 5:** Hierarchical regression analysis: predicting perceived enjoyment.

Step and predictor	Leisure context (*n* = 186)		Work context (*n* = 182)	
	Step 1 β	Step 2 β	Step 1 β	Step 2 β
(1) Perceived usefulness	0.66^∗∗∗^	0.61^∗∗∗^	0.67^∗∗∗^	0.64^∗∗∗^
Perceived ease of use	0.23^∗∗∗^	0.17^∗∗^	0.17^∗∗^	0.13^∗^
(2) Subjective norms		0.10^∗^		0.13^∗^
Personal innovativeness		0.10^∗^		0.03
*R*^2^	0.630	0.647	0.554	0.570
Δ*R*^2^	0.630	0.017	0.554	0.017
*F*_change_	155.90^∗∗∗^	4.41^∗^	110.99^∗∗∗^	3.42^∗^
*Degree of change freedom*	(2,183)	(2,181)	(2,179)	(2,177)

*^∗^*p* < 0.05, ^∗∗^*p* < 0.01, ^∗∗∗^*p* < 0.001.*

As shown in **Table 5**, in step 1, the two original TAM variables resulted in substantial and significant counts, representing 63.0% and 55.4% of the observed variance in the leisure and work contexts (*F* ≥ 110.99, *p* < 0.001), respectively. Perceived usefulness (β ≥ 0.66, *p* < 0.001) and PEU (β ≥ 0.17, *p* < 0.01) emerged as very significant predictors in both contexts. For both contexts, as presented in step 2, the extended TAM variables, when added to the regression equation, only increased the variance by an additional 1.7%, and subjective norms emerged as a weak significant predictor (β ≥ 0.10, *p* < 0.05) (*F* ≥ 3.42, *p* < 0.05). However, personal innovativeness tended to be a weak significant predictor in the leisure context only (β = 0.10, *p* < 0.05). All of these findings indicated that perceived usefulness had a greater influence on mobile video calling than PEU. Furthermore, there were limited differences in the degree to which various factors affected behavioral intentions for different contexts (the work and leisure contexts), except for the influence of personal innovativeness.

## Discussion

The goals of this research were to examine the factors influencing users’ intention to use video calling (especially the effect of perceived enjoyment on users’ intention to adopt mobile video calling), as well as to distinguish the influence of different contexts (the work context and the leisure context) on predicting user acceptance of this service. The data collected from participants were utilized to test the proposed research model. The results showed that users’ intentions were directly determined by perceived usefulness and perceived enjoyment, and the proposed research model explained 56.0% and 61.5% of the total variance in users’ intentions in the work and leisure contexts, respectively. In addition, there were some context-specific differences in predicting user acceptance of mobile video calling.

### Predicting Effects of Perceived Usefulness and Perceived Enjoyment on Video Calling Use Decisions

From the perspective of user acceptance, we examined the effects of perceived usefulness, PEU, perceived enjoyment, subjective norms, and personal innovativeness on mobile video calling via the proposed research model. The results of this research indicated that perceived enjoyment could be considered a core predictor along with perceived usefulness in predicting users’ intentions toward mobile video calling, especially for the leisure context.

In line with our expectations, hypotheses 1 and 3 were confirmed. Perceived enjoyment and perceived usefulness emerged as significant predictors of the intention to use mobile video calling. This finding is consistent with the findings of prior TAM-based studies (e.g., Lu and Xu, 2006) indicating that the predictive effect of perceived enjoyment on behavioral intention is direct. Additionally, our results are in agreement with those of prior TAM-based studies of ICT, indicating that perceived usefulness is a strong predictor of acceptance intention in TAM (Davis, 1989). In general, perceived usefulness is a cognitive belief, while perceived enjoyment tends to reflect users’ feelings in both pre-acceptance and post-acceptance. Although the predictive power of these two factors (perceived usefulness and perceived enjoyment) varies across contexts, the associations between them and video call users’ acceptance are strong.

Regarding practical implications, perceived enjoyment, as an added factor in the basic structure, exerted a significant effect on users’ intention to use mobile video calling in both contexts. Therefore, to enhance consumers’ behavioral intention to adopt mobile value-added services, such as video calling, perceived enjoyment should be the primary focus. This finding suggests that in a specific context, users’ motivation to use a mobile value-added service is derived from the enjoyment of using the service. Mobile video calling services offer visual and interactive functions, including the convenience of enabling people on both sides to watch dynamic images on the screen and experience the good feelings that result from communicating. Furthermore, these services offer the benefit of being cost-effective for potential customers. These rich features of mobile value-added service can provide customers with a more pleasant experience. Therefore, the entertaining and enjoyable content provided by video calling services can be treated as an important factor determining the acceptance of this service. In summary, to enhance consumers’ behavioral intention to use video calling, providers do not need to convey a great deal of technical information to consumers when promoting value-added services; instead, they should consider focusing on improving consumers’ perceived enjoyment of this technology.

### Predicting Effects of Related Variables on Perceived Enjoyment

The results of the current study show that the perceived enjoyment of using video calling is significantly affected by perceived usefulness, indicating that users’ perception of usefulness is a key determinant of perceived enjoyment levels, which is in line with hypothesis 1. Thus, according to hypothesis 4, perceived usefulness influences intention in two ways: directly and indirectly via perceived enjoyment. Furthermore, PEU is positively related to perceived enjoyment. These findings are consistent with hypothesis 2. In addition, subjective norms emerged as a weak significant predictor of perceived enjoyment in both the work context and the leisure context. This finding supports hypothesis 4 to some extent. Venkatesh et al. (2003) found that subjective norms significantly influenced the behavioral intention of accepting information technology in a mandatory environment (i.e., the work context). In this study, subjective norms emerged as a significant predictor of use intention only in the leisure context. This finding may be related to the characteristics of video calling. People tend to be more engaged in video calling in the leisure context than in the work context. Communicating with friends in a face-to-face way makes users perceive greater enjoyment. If users realize that many people are using video calling, this may increase users’ willingness to use video calling. However, personal innovativeness tended to be a weak significant predictor of both users’ acceptance and perceived enjoyment in the leisure context only. Thus, hypothesis 6 was partially supported. This finding indicated that people who reported high personal innovativeness tended to report high use intention and enjoyment in the leisure goal context.

According to our research, the improvement of perceived enjoyment depends on increases in perceived usefulness, PEU and subjective norms in general. Providers attempting to attract consumers to use this service can increase users’ satisfaction by improving the usefulness of the technology and by making the video call interface easier to operate, both of which can directly affect users’ behavioral intention to adopt video calling. Additionally, improving public perceptions of the technology and encouraging more users to adopt video calling services may cause users to perceive greater social approval from important others. Thus, consumers will be more willing to use this technology and obtain more enjoyment when doing so.

### Effects of Different Contexts on Predicting User Acceptance

In the current study, perceived enjoyment was the most important predictor of users’ intention to use video calling in the leisure context, while perceived usefulness was the most important predictor in the work context. Additionally, perceived enjoyment and perceived usefulness had different roles regarding the context. Our results revealed that context-specific differences influenced users’ video calling acceptance, which supports hypothesis 7. For example, perceived usefulness and perceived enjoyment jointly determined the behavioral intention to use video calling in both the work context and the leisure context. However, in the work context, the role of perceived usefulness was more important for explaining users’ behavioral intentions than perceived enjoyment was. By contrast, perceived enjoyment had a more powerful effect on behavioral intentions in the leisure context. In addition, the predictive effect of perceived usefulness on perceived enjoyment was stronger in the work context than in the leisure context. However, the effect of PEU on perceived enjoyment was weaker in the work context than in the leisure context. These important findings indicate the necessity of distinguishing between different contexts (the work context and the leisure context) when predicting user acceptance of video calling. These results are consistent with those of previous studies (Moon and Kim, 2001; Bruner and Kumar, 2005). Users’ attitudes can be influenced by situational factors and by the interaction between the individual and his or her situation.

Thus, to attract more consumers to video calling services and to reduce the cost of developing video calling technology, providers in the telecommunications industry should consider developing two different usage context patterns for video calling. Technology development could be better targeted to consumers’ different demands in different contexts. For instance, the effect of perceived enjoyment was greater in the leisure context than in the work context. Therefore, developers should pay more attention to the recreational design of video calling, adding more attractive entertainment elements when developing technology for the leisure context. In interactive video chat, for example, they might add the effect of a dynamic figure to express the behavior of kissing by showing a cartoon kiss in the video call screen. By contrast, providers should devote more attention to improving the practicality of video calling technology and help users improve task performance for the work context, where perceived usefulness is more important. Additionally, the work context design may need to be more formal in appearance. Because the video call interface is easy to operate on users’ mobile devices, users are not overly concerned about PEU in the work context. However, in the leisure context, users consider PEU: the less effort users need to expend in learning to use video calling, the more willing they will be to use it in the leisure context.

## Conclusion and Limitations

This study investigated consumers’ behavioral intentions to adopt a specific mobile value-added service: video calling. The following conclusions were reached. First, as the results of this study show, perceived enjoyment is one important factor directly impacting consumers’ behavioral intention to adopt video calling technology. The perceived enjoyment of using video calling is significantly affected by perceived usefulness, followed by PEU. In particular, the predictive effect of perceived usefulness on perceived enjoyment is stronger in the work context than that in the leisure context. However, the effect of PEU on perceived enjoyment is weaker in the work context than in the leisure context. Second, the intention to use video calling services is primarily determined by perceived usefulness and perceived enjoyment. In the work context, perceived usefulness has a stronger predictive effect on the intention to use video calling. By contrast, perceived enjoyment has a more powerful effect on behavioral intention in the leisure context. Third, as external variables, subjective norms and personal innovativeness both have a very limited influence on perceived enjoyment and on the intention to use video calling. Finally, the degree of the effect exercised by all of the reliable factors on the intention to use video calling differs in different contexts. Users in the leisure context expressed a stronger intention to use video calling services, in addition to demanding greater ease of use and a higher level of enjoyment in using value-added video calling services, compared with users in the work context.

The limitations of this study are as follows. First, the subjects were university students from Beijing and Beihai, China, which means that the results could not be generalized to all consumers. Second, unlike in Western countries, the 4G telecom service market in China is still under development and the costs are relatively high. Thus, a sampling of all the practical users of mobile value-added services was not available. Third, as a result of time constraints, only cross-sectional data were analyzed. The extent to which current behavioral intention to use video calling can be used to predict future behavior is unknown. Because a longitudinal research method could not be adopted, prudence was required in the discussion of causal relationships between constructs. In the future, when consumers have a higher level of involvement in mobile value-added services, studies in this field could undertake in-depth investigations to allow more objective conclusions. Additionally, this study primarily investigated the endogenous variables (and a few external variables) affecting behavioral intentions. Follow-up studies could examine more external variables affecting the adoption of mobile value-added services, such as personality traits. External variables, especially personal innovativeness, should be considered along with a combination of personal traits. For instance, personality traits (such as the Big Five) could also be relevant important predictors in determining users’ social network sites, which are a typical interpersonal technology (e.g., Chuang et al., 2017). To obtain more comprehensive data, the sampled user groups could be enlarged, and differences in behavioral intentions between different user groups could be compared.

## Author Contributions

Conceived and designed the experiments: RZ. Performed the survey: RZ. Analyzed the data: RZ. Wrote the manuscript: RZ and CF.

## Conflict of Interest Statement

The authors declare that the research was conducted in the absence of any commercial or financial relationships that could be construed as a potential conflict of interest.
